# Screening of a Gossypol-Removing Yeast Strain and Characterization of Its Removal Mechanism

**DOI:** 10.3390/microorganisms13102251

**Published:** 2025-09-25

**Authors:** Yushuo Zhang, Tingyao Lv, Qiuyang Jiang, Xiaotong Zeng, Feng Li, Dayong Xu

**Affiliations:** 1 Anhui Province Key Laboratory of Pollutant Sensitive Materials and Environmental Remediation, Huaibei Normal University, Huaibei 235000, China; 15562580727@163.com (Y.Z.);; 2 School of Life Sciences, Huaibei Normal University, Huaibei 235000, China

**Keywords:** gossypol, yeast, screening, solid-state fermentation, removal mechanism

## Abstract

Gossypol, a polyphenolic naphthalene derivative and yellow polyphenolic pigment found in cotton seed glands, presents notable environmental, animal, and human health hazards. To screen for yeast strains capable of utilizing gossypol and to investigate their removal efficiency and mechanisms. Yeast strains capable of utilizing gossypol as the exclusive carbon source were isolated from cotton field soil. The identification of these strains involved assessment of colony morphology, physiological and biochemical characteristics, and phylogenetic analysis utilizing 26S rDNA gene sequences. Safety evaluations included hemolytic and antibiotic susceptibility tests. The growth responses of the selected strains to varying temperatures and pH levels were determined. Using cotton meal as the solid fermentation substrate, the effects of single factors on gossypol removal by the strains were determined. The intracellular and extracellular localization as well as the nature of the gossypol-removing active components in the strains were characterized, followed by an investigation into the molecular mechanism of gossypol removal using LC-MS analysis. A total of 17 gossypol-utilizing strains were isolated from cotton field soil samples, with strain ZYS-3 demonstrating superior removal capability. Strain ZYS-3 was identified as *Meyerozyma guilliermondii*, exhibiting no hemolytic activity and susceptibility to nine commonly used antifungal agents. The optimal growth parameters for this strain were determined to be a temperature of 30 °C and a pH of 5.0. In solid-state fermentation using cotton meal at 30 °C with initial fermentation conditions (10% corn flour added as an external carbon source, 40% moisture content, and 6% inoculum concentration) for 3 days, strain ZYS-3 achieved a gossypol removal rate of 73.57%. Subsequent optimization of the fermentation process, including the addition of 10% corn flour as an external carbon source, adjustment of moisture content to 55%, and inoculum concentration to 10%, resulted in an increased gossypol removal rate of 89.77% after 3 days of fermentation, representing a 16.2% enhancement over the initial conditions. Assessment of gossypol removal activity revealed that strain ZYS-3 predominantly removes gossypol through the secretion of extracellular enzymes targeting specific active groups (phenolic hydroxyl groups and aldehyde groups) within the gossypol molecule. These enzymes facilitate oxidation and elimination reactions, leading to the opening of the naphthalene ring and subsequent removal of gossypol.

## 1. Introduction

Gossypol (C_30_H_30_O_8_) is a polyphenolic di-naphthalene derivative predominantly present in cotton seeds, particularly in cotton seed kernels [1,2]. With a molecular weight of 518, gossypol is characterized by the chemical structure 2,2′-bis(8-formyl-1,6,7-trihydroxy-5-isopropyl-3-methylnaphthalene). It exhibits insolubility in water and hexane but solubility in acetone, chloroform, ether, and butanone, and is partially soluble in crude vegetable oil [3,4,5].

Gossypol primarily exists in two forms: free gossypol and bound gossypol. Bound gossypol, which is not absorbed in the animal digestive tract and is promptly excreted in feces, exhibits relatively low toxicity. Conversely, free gossypol can be absorbed by the gastrointestinal tract of animals, with its active hydroxyl and aldehyde groups demonstrating significant cytotoxicity, reproductive toxicity, and oxidative stress-inducing effects. This results in growth inhibition, organ damage, and reproductive disorders in monogastric animals (such as pigs, poultry, and aquatic animals), posing a direct threat to food safety from animal-derived products due to residual concerns [6,7,8,9]. Therefore, effectively removing or removing free gossypol in cottonseed meal is essential for enhancing its feed quality, safeguarding animal health, and optimizing resource utilization efficiency.

Currently, the primary approaches for detoxifying gossypol encompass physical methods [10,11], chemical methods [12], and biological methods. Biological methods are recognized as the most effective means for eliminating free gossypol from cottonseed meal and enhancing its nutritional quality. Among biological methods, microbial-based treatments are cost-effective, safe, and have the potential to elevate the protein and essential amino acid levels in cottonseed meal, thereby enhancing feed attractiveness. Consequently, microbial treatments have gained traction for gossypol detoxification in cottonseed meal in recent years [13]. Despite reports indicating the gossypol-removing capabilities of select microorganisms, there remains a scarcity of microbial strains that exhibit efficient, stable, and safe gossypol removal properties. Many industrial yeast strains are commonly utilized in the feed sector due to their generally recognized as safe (GRAS) status. It synthesizes a diverse array of enzymes that efficiently degrade complex molecules in feed, promote feed conversion, and improve feed utilization [14]. Additionally, the identification of degradative enzymes in biological methods has been a focal point in the removal of analogous deleterious substances. Enzymes exhibit notable efficacy and safety in gossypol removal; however, research on gossypol-removing enzymes remains limited both domestically and internationally, necessitating further strides in the discovery and optimization of degradative enzymes.

Therefore, this study aims to isolate and screen a yeast strain capable of efficiently removing free gossypol, conduct a comprehensive assessment of its biosafety and growth characteristics, optimize the detoxification process parameters in a cotton meal solid-state fermentation system, and elucidate the molecular mechanism of gossypol removal by identifying the intracellular and extracellular localization of the active constituents engaged in gossypol removal. The findings of this study are anticipated to establish a foundational framework for utilizing strain ZYS-3 as a detoxification feed inoculant for cottonseed meal and for the genetic exploration and modification of gossypol-removing enzymes.

## 2. Materials and Methods

### 2.1. Materials

#### 2.1.1. Sample Collection

The yeast strains utilized in this study were sourced from cotton fields in Zhangqiu District, Jinan City, Shandong Province. Soil samples were gathered from the uppermost 3–10 cm layer, which comprises remnants of cotton plants and leaf litter.

#### 2.1.2. Culture Media

Yeast Screening Medium: glycerol 5.0 g, cotton meal 2.0 g, KH_2_PO_4_ 0.5 g, (NH_4_)_2_SO_4_ 1.0 g, yeast extract 0.5 g, in 1000 mL water, pH unadjusted.

YPD liquid medium: glucose 20 g, peptone 20 g, yeast extract 10 g, in 1000 mL water, pH unadjusted.

YPD solid medium: YPD liquid medium supplemented with 2% agar.

GAMM liquid medium (With gossypol as the sole external carbon source): Gossypol 0.6 g, NaCl 1.0 g, (NH_4_)_2_SO_4_ 5.0 g, MgSO_4_·7H_2_O 0.5 g, KH_2_PO_4_ 1.0 g, in 1000 mL water, pH unadjusted.

GAMM solid medium (with gossypol as the sole external carbon source): GAMM liquid medium supplemented with 2% agar.

Columbia blood agar medium: Columbia blood agar powder 40.0 g, sodium chloride 5 g, in 1000 mL water.

#### 2.1.3. Main Materials and Instruments

Test Materials: Cotton seed meal sourced from Hebei Cotton Seed Meal Processing Plant; Gossypol procured from Shanghai McLean Bio-Chemical Technology Co., Ltd. (Shanghai, China); Fungal Antimicrobial Susceptibility Test Kit DL-96 Fungi from Zhuhai Dier Bio-Engineering Co., Ltd. (Zhuhai, China); Yeast Identification Test Kit API 20 C AUX supplied by Merieux Diagnostics Products (Shanghai) Co., Ltd. (Shanghai, China).

Test Instruments: PHB-10 Pen-Type pH Meter by Shengong Bioengineering (Shanghai) Co., Ltd. (Shanghai, China); MQL-61 R Oscillating Incubator manufactured by Shanghai Yiquan Instrument Co., Ltd. (Shanghai, China); Ultimate3000 High-Performance Liquid Chromatograph by Dionex Corporation (Sunnyvale, CA, USA); Digital Temperature-Controlled Water Bath from Qun’an Test Instrument Co., Ltd. (Huzhou, China); Clean Bench provided by Shanghai Boxun Medical and Biological Instrument Co., Ltd. (Shanghai, China); SQ810 C Vertical Pressure Steam Sterilizer by Chongqing Yamato Technology Co., Ltd. (Chongqing, China); L4-5 K Desktop Low-Speed Centrifuge from Hunan Kecheng Instrument Equipment Co., Ltd. (Changsha, China); UV-4802 UV-Vis Spectrophotometer by Shanghai Unico Instrument Co., Ltd. (Shanghai, China).

### 2.2. Screening of Gossypol-Removing Yeast Strains

#### 2.2.1. Preliminary Screening of Gossypol-Removing Yeast Strains

1 g of the experimental soil sample was weighed and introduced into a liquid culture medium designed for Gossypol-removing yeast enrichment. The mixture was incubated at 30 °C and 220 rpm for 7 days.

The enriched solution was diluted, spread onto GAMM solid medium, and then incubated at 30 °C for 3–4 days. Upon the formation of colonies, yeast-like colonies were selected, isolated, and purified onto YPD solid medium, followed by incubation at 30 °C for 3–4 days.

#### 2.2.2. Rescreening of Gossypol-Removing Strains

The isolated and screened strains capable of utilizing gossypol as the sole external carbon source were cultured in a liquid medium containing gossypol as the sole external carbon source at a final concentration of 600 µg/mL. The culture was maintained at 30 °C and 220 rpm for 5 days. Subsequent to incubation, the yeast cell suspension was mixed with acetone at a 1:3 ratio, followed by centrifugation at 10,000 rpm for 5 min. The resulting supernatant was filtered through a 0.22 µm organic-based filter membrane to obtain the test sample [15]. The residual gossypol content was detected using high-performance liquid chromatography (HPLC) with a YMC-PACK ODS series C18 column (Kyoto, Japan) (dimensions: 250 mm length × 4.6 mm inner diameter × 2.6 µm particle size). The mobile phase A consisted of acetonitrile: 0.2% phosphoric acid (volume ratio 83:17) at a flow rate of 1 mL/min, with an injection volume of 20 µL. The column temperature was maintained at 25 °C, and detection was performed using a UV detector set at a wavelength of 238 nm.

### 2.3. Strain Identification and Physiological and Biochemical Characteristic Testing

Morphological Characterization: The isolated yeast strains were initially identified through plate observation, optical microscopy, and electron microscopy.

26S rDNA Sequence Determination and Analysis: The fungal strains were grown to the stationary phase, and genomic DNA was extracted. This DNA was used as the template for PCR amplification of the 26S rDNA D1/D2 region with the universal primers NL1 (5′-GCATATCAATAAGCGGAGGAAAAG-3′) and NL4 (5′-GGTCCGTGTTTCAAGACGG-3′) [16]. The amplified products were sent to a sequencing company for sequencing. The obtained sequences were analyzed by BLAST in the GenBank database, and A phylogenetic tree was constructed using MEGA 11.0 software with the Neighbor-Joining method (Construct/Test Neighbor-Joining tree).

Physiological and Biochemical Properties: The strains were subjected to physiological and biochemical assessments using a yeast identification kit (API 20 C AUX, bioMérieux, Marcy-l’Étoile, France).

### 2.4. Safety Evaluation

#### 2.4.1. Hemolysis Test

A single colony of strain ZYS-3 was streaked onto Columbia blood agar plates, with pathogenic *Staphylococcus aureus* as the positive control. The plates were incubated upside down at 30 °C for 48 h, followed by observation for the presence of hemolytic zones.

#### 2.4.2. Antifungal Susceptibility Testing

The DL-96 Fungi antifungal susceptibility test kit (Zhuhai Deer Bioengineering Co., Ltd., Zhuhai, China) was employed for the experiment. The ZYS-3 strain was cultured on YPD plates for 24 h, and multiple single colonies were picked and adjusted to a yeast suspension of 0.5 McFarland units. Initially, 100 µL of fungal susceptibility solution was added to well H12 of the test plate as a negative control. Subsequently, 20 µL of the yeast suspension was combined with the fungal susceptibility solution, thoroughly mixed, and 100 µL was dispensed into each well of the test plate. Following incubation at 35 °C for 48 h, observations were conducted to identify any pink or purple coloration in the positive control well.

### 2.5. Effect of Different Culture Conditions on Strain Growth

The optimal growth conditions for strain ZYS-3 were determined by measuring OD_600_ values at different temperatures, pH levels, and time points. First, the strain was inoculated into YPD medium (1% inoculation ratio) and cultured at temperatures ranging from 20 to 45 °C (in 5 °C increments) and pH values from 4.0 to 10.0 for 24 h, followed by OD_600_ measurement to determine the optimal temperature and pH. Subsequently, under the optimized temperature and pH conditions, samples were collected every 3 h to measure OD_600_ and plot the growth curve. All cultures were incubated in a shaking incubator at 220 rpm, and OD_600_ values were measured using an ultraviolet spectrophotometer.

### 2.6. Single-Factor Optimization of Strain Removal of Gossypol in Cotton Meal Substrate

With the optimal temperature for strain ZYS-3 identified as 30 °C, 50 g of fermentation substrate (comprising 45 g cotton seed meal and 5 g corn flour) was placed in a fermentation bag for solid-state fermentation under initial fermentation conditions (40% moisture content, 6% inoculum, and 10% corn flour supplement as an external carbon source). Samples were collected at 24 h, 48 h, 72 h, 96 h, and 120 h of solid-state fermentation, and the gossypol removal rate was determined. A relationship curve between fermentation time (*x*-axis) with gossypol removal rate (*y*-axis) was plotted to determine the optimal fermentation time for cotton seed meal.

After determining the optimal fermentation time, 45 g of fermentation material (comprising 45 g cotton seed meal and 5 g corn flour) was placed in a fermentation bag. Glucose, corn flour, starch, and sucrose were introduced at a 10% ratio as external carbon sources for solid-state fermentation. Subsequent to fermentation, samples were collected, and the gossypol removal rate was assessed. The fermentation effects under various carbon sources were compared by plotting the gossypol removal rate and live yeast count against different carbon sources to identify the optimal carbon source for the strain’s fermentation.

After establishing the optimal fermentation time and optimal external carbon source, 45 g of fermentation material (45 g cotton seed meal + 5 g corn flour) was placed in a fermentation bag. Water was added at moisture contents of 40%, 45%, 50%, 55%, and 60% for solid-state fermentation. Following fermentation, samples were collected, and the gossypol removal rate was measured. A relationship curve correlating moisture content with gossypol content and gossypol removal rate was plotted, with moisture content on the *x*-axis and gossypol parameters on the *y*-axis, to determine the optimal moisture content for cottonseed meal fermentation.

Following the determination of the optimal fermentation time, carbon source, and moisture content, 45 g of fermentation material (45 g of cotton seed meal + 5 g of corn flour) was placed in a fermentation bag and inoculated with yeast solution at ratios of 2%, 4%, 6%, 8%, and 10% for solid-state fermentation. After fermentation, samples were collected, and the gossypol removal rate was quantified. A relationship curve was constructed, with the inoculation amount on the *x*-axis and gossypol parameters on the *y*-axis, to identify the optimal inoculation amount for cottonseed meal fermentation.

### 2.7. Determination of the Intracellular and Extracellular Localization and Types of Active Components Involved in the Removal of Gossypol by Strain ZYS-3

The yeast suspension of strain ZYS-3 was inoculated at a 1% volume fraction into YPD medium containing 600 µg/mL gossypol. The culture was incubated at 30 °C and 220 rpm for 9 days until complete gossypol removal. Subsequently, four samples were prepared by centrifuging the yeast suspension at 5000 rpm/min for 10 min: 1. The first sample involved collecting the supernatant from the yeast culture, filtering it through a 0.22 µm water-based filter membrane for future use; 2. The second sample entailed collecting the supernatant from the yeast culture, filtering it through a 0.22 µm water-based filter membrane, and sterilizing it in an autoclave at 121 °C for 20 min for later use; 3. For the third sample, the supernatant of the yeast suspension was discarded, the yeast pellet was retained, resuspended in sterile water, and sterilized in an autoclave at 121 °C for 20 min for future use; 4. In the fourth sample, the supernatant of the yeast suspension was decanted, the yeast pellet was retained, resuspended in sterile water and subjected to cell disruption using an ultrasonic cell disruptor. The cell disruption solution was centrifuged at 12,000 rpm for 30 min, and the supernatant of the disrupted yeast cells was filtered through a 0.22 µm membrane and reserved for subsequent use. Gossypol at a concentration of 200 µg/mL was introduced to the samples, followed by a 6 h incubation period. The gossypol residue (peak area) was measured. A control using YPD medium without the ZYS-3 strain was employed to calculate the gossypol removal rate.

Based on the highest removal rate observed in the prior experiments, two additional sets of experiments were designed. The first sample involved preparing a ZYS-3 yeast suspension devoid of gossypol induction to assess the potential production of gossypol-removing substances in the absence of gossypol induction. The second sample entailed the addition of proteinase K (1 mg/mL) to disrupt proteins, aiming to investigate the contribution of protein-based substances in gossypol removal. Both samples were incubated with gossypol (200 µg/mL) for 6 h, and the gossypol content was quantified.

### 2.8. Detection of Gossypol Removal Products

The supernatant from strain ZYS-3 served as the control group, while the supernatant from the strain incubated with gossypol for 6 h was designated as the experimental group. A 600 µL portion of the fermentation broth was combined with 600 µL of acetonitrile, filtered through a 0.22 µm organic phase filter membrane, and subsequently injected into the LC-MS system for the detection of removal intermediates.

The LC gradient elution program was set as follows: 0–5 min, 50% acetonitrile/50% 0.1% formic acid; 5–8 min, linear gradient from 50% to 100% acetonitrile (corresponding decrease of 0.1% formic acid from 50% to 0%); 8–12 min, 100% acetonitrile/0% 0.1% formic acid; 12–15 min, linear gradient from 100% to 5% acetonitrile (corresponding increase of 0.1% formic acid from 0% to 95%). The column temperature was held at 40 °C. Detection was performed at 375 nm. The flow rate was 0.4 mL/min with an injection volume of 5 μL.

For MS detection, electrospray ionization was operated in negative mode. The mass scan range was set to *m*/*z* 50–1000. The fragmentor voltage was 200 V. Other parameters were set to default values.

## 3. Results and Discussion

### 3.1. Results of Screening, Isolation and Purification of Strains

**Figure 1:**
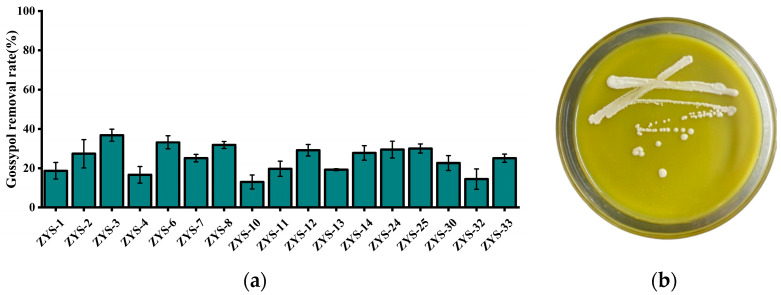
Results of strain screening, isolation and purification. (**a**) Gossypol removal rates by 17 isolated strains in GAMM liquid medium; (**b**) Growth status of strain ZYS-3 on solid medium plates with gossypol as the sole supplemental carbon source.

Seventeen yeast-like strains capable of utilizing gossypol as the sole external carbon source were isolated, screened, and purified from soil samples. Notably, strain ZYS-3 demonstrated robust growth on solid medium with gossypol as the sole external carbon source (Figure 1b) and exhibited the most efficient gossypol removal in liquid medium with gossypol as the sole external carbon source (Figure 1a). Therefore, strain ZYS-3 was selected for subsequent investigations.

### 3.2. Strain Identification Results

**Figure 2:**
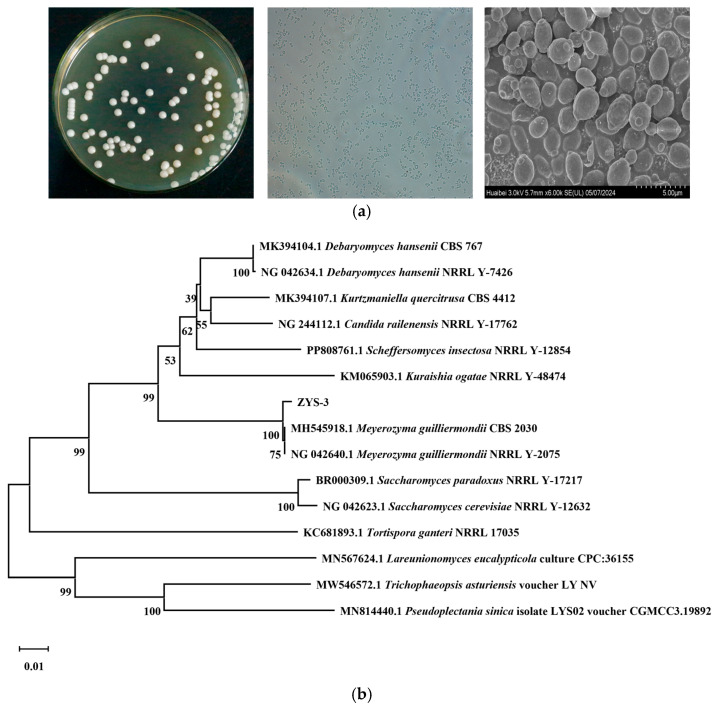
Morphological characteristics of strains and phylogenetic tree. (**a**) Plate observation results, optical microscope observation results, and electron microscope observation results. (**b**) Phylogenetic tree construction.

Strain ZYS-3 forms round colonies on YPD plates, with an average diameter of approximately 4.0 mm. The colonies are characterized by a creamy white color, a convex center, smooth and glossy surface, moist texture, and well-defined edges. Microscopically, the cells appear predominantly oval-shaped, ranging in size from 0.4 μm to 0.6 μm. Ultrastructural examination using an electron microscope reveals oval-shaped cells arranged irregularly, displaying distinct bud scars (Figure 2a).

The 26S rDNA D1/D2 region gene sequence of this strain was amplified, sequenced, and utilized to construct a phylogenetic tree for analysis (Figure 2b). ZYS-3 clustered with *Meyerozyma guilliermondii*, indicating a high phylogenetic affinity. Consequently, ZYS-3 is classified as *M guilliermondii* ZYS-3. (The sequence alignment results and the annotation of the 26S rDNA target region are provided in the Supplementary Materials). This strain has been deposited at the China Typical Culture Collection Center under the accession number CCTCC M 20251701.

Metabolic characteristics were analyzed using the yeast identification kit API 20 C AUX. The results showed that strain ZYS-3 exhibited positive reactions to glucose, glycerol, 2-ketoglutarate hydrochloride, arabinose, xylose, chrysanthemum alcohol, xylitol, galactose, sorbitol, α-methyl-D-glucose, acetyl-glucosamine, cellobiose, maltose, sucrose, trehalose, xylose, and raffinose. Conversely, negative reactions were observed for inositol and lactose (Table 1).

### 3.3. Safety Evaluation Results of Strain ZYS-3

#### 3.3.1. Hemolytic Activity

Hemolysis refers to the rupture of red blood cells and subsequent hemoglobin release induced by hemolytic toxins and various physical and chemical factors. This process is pivotal in the pathogenesis of animal diseases. The assessment of hemolytic activity in yeast metabolic products serves as a crucial parameter for evaluating the safety profile of yeast strains.

**Figure 3:**
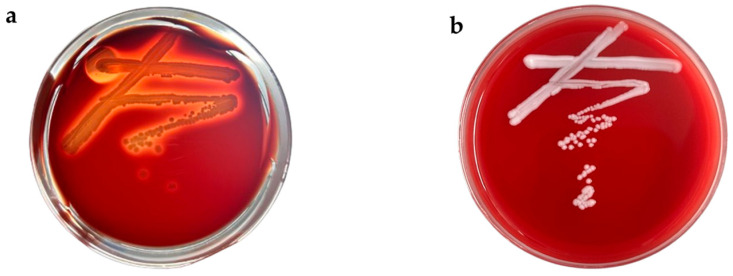
Hemolysis on Columbia blood agar plates. (**a**) Hemolysis results for Staphylococcus aureus. (**b**) Hemolysis results for ZYS-3 yeast.

The test strain was inoculated onto Columbia blood agar plates, and the appearance of clear zones was observed (Figure 3b). Notably, strain ZYS-3 did not form green hemolytic zones or clear hemolytic zones on the blood agar plates, in contrast to the distinct clear hemolytic zones produced by Staphylococcus aureus (Figure 3a). These findings indicate that strain ZYS-3 does not produce hemolytic toxins, leading to its preliminary classification as a safe strain.

#### 3.3.2. Antifungal Resistance

The outcomes of the antifungal susceptibility testing kit are shown in Table 2. The study encompassed the assessment of nine frequently employed antifungal medications: amphotericin B, flucytosine, micafungin, caspofungin, fluconazole, Ethanol, voriconazole, posaconazole, and itraconazole.

The screened strain ZYS-3 demonstrated sensitivity (S) to all nine drugs without displaying any resistance, indicating that strain ZYS-3 is devoid of drug resistance concerns, thereby ensuring its safety profile.

### 3.4. Growth Characteristics of Strain ZYS-3 Under Different Conditions

**Figure 4:**
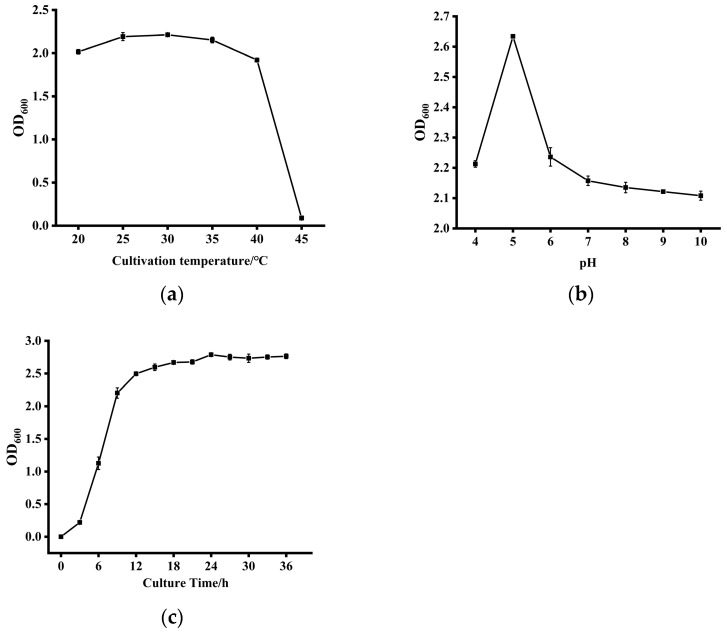
Culture conditions for strain ZYS-3. (**a**) Effect of different temperatures on the growth of strain ZYS-3; (**b**) Effect of different pH levels on the growth of strain ZYS-3; (**c**) Growth curve of strain ZYS-3.

As depicted in Figure 4a, strain ZYS-3 demonstrates robust stability in growth and reproductive capacity across the temperature spectrum of 20 °C to 40 °C, with marginal variations in growth performance observed at different temperature levels. Nevertheless, a significant decline in the OD_600_ value is noticeable upon exceeding 40 °C, indicating a pronounced inhibition of growth and reproduction. Therefore, the optimal growth temperature for strain ZYS-3 is approximately 30 °C.

As illustrated in Figure 4b, under optimal conditions at 30 °C, strain ZYS-3 exhibits OD_600_ values ranging notably from 2.097 to 2.637 across pH levels from 4 to 10. Notably, the highest OD_600_ value of 2.637 is observed at pH 5, signifying pH 5 as the most conducive growth condition.

As shown in Figure 4c, the growth curve analysis of strain ZYS-3 under optimal conditions (30 °C, pH 5) indicates a lag phase from 0 to 3 h, followed by a vigorous logarithmic growth phase between 3 and 9 h. After 9 h, the growth rate decreases and transitions into the stationary phase.

### 3.5. Optimization Results of Single-Factor Conditions for Solid-State Fermentation Using Cotton Seed Meal as Substrate

The removal rate of gossypol by strain ZYS-3 in cotton seed meal under various conditions was assessed to simulate the gossypol removal process during solid-state fermentation.

**Figure 5:**
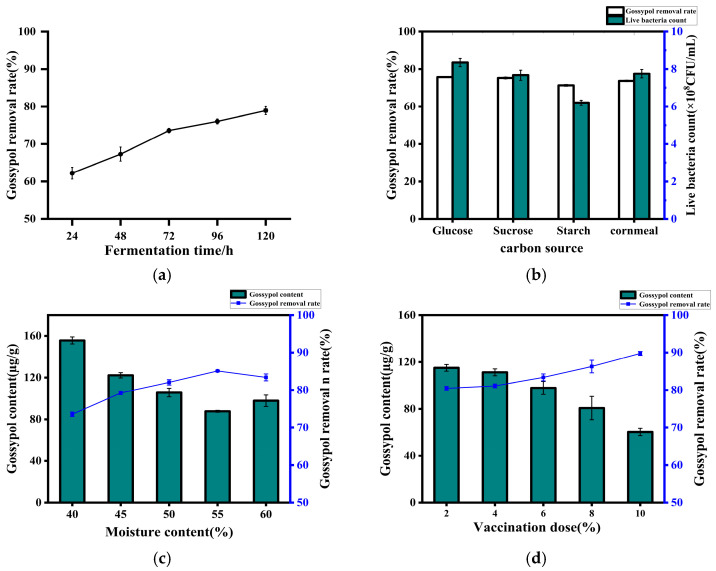
Removal rate of gossypol by strain ZYS-3 under solid-state fermentation. (**a**) Effect of solid-state fermentation time on the removal of gossypol by strain ZYS-3. (**b**) Effect of added carbon source on the removal of gossypol by strain ZYS-3. (**c**) Effect of moisture content on the removal of gossypol by strain ZYS-3. (**d**) Effect of inoculum amount on the removal of gossypol by strain ZYS-3.

As depicted in Figure 5a, the gossypol content in cotton meal gradually decreased with increasing fermentation time. Notably, compared to the 96 h and 120 h fermentation durations, cotton meal fermented for 72 h exhibited a rich yeast-like sweet aroma with no off-odors. The material displayed a moderate viscosity, and the cotton seed extract removal rates at 72 h and 120 h did not show significant differences. Considering both palatability and fermentation efficiency, 72 h was identified as the optimal fermentation duration.

As shown in Figure 5b, the removal rates of glucose, sucrose, starch, and corn flour (utilized as carbon sources) were 75.64%, 75.14%, 71.24%, and 73.57%, respectively. Following a 3-day fermentation, the live yeast counts in fermented cotton seed meal supplemented with these four carbon sources (glucose, sucrose, starch, and corn flour) was 8.35 × 10^8^ CFU/g, 7.67 × 10^8^ CFU/g, 6.2 × 10^8^ CFU/g, and 7.75 × 10^8^ CFU/g, respectively. The marginal discrepancies observed could be attributed to the inherent presence of sugars, proteins, and amino acids in cotton seed meal, which may mitigate the influence of added carbon sources on yeast proliferation [17]. Based on considerations of cost-effectiveness, corn flour was selected as the added carbon source.

As illustrated in Figure 5c the strain’s efficiency in gossypol removal is positively correlated with the moisture content of the material. However, once the moisture content surpasses 55%, the gossypol removal rate shows a declining trend. This observation may be attributed to excessive moisture causing clumping of the cotton meal, hindering adequate oxygen diffusion within the material and consequently impacting the strain’s gossypol removal efficiency [18,19]. Conversely, insufficient moisture content can lead to sluggish yeast growth and limited reproduction. Therefore, 55% moisture content was determined as the optimal level.

As depicted in Figure 5d, the efficiency of gossypol removal demonstrates a positive correlation with the inoculum concentration, peaking at 89.77% with a 10% inoculum concentration.

### 3.6. Results on the Intracellular and Extracellular Localization and Types of Active Components Responsible for Gossypol Degradation by Strain ZYS-3

**Figure 6:**
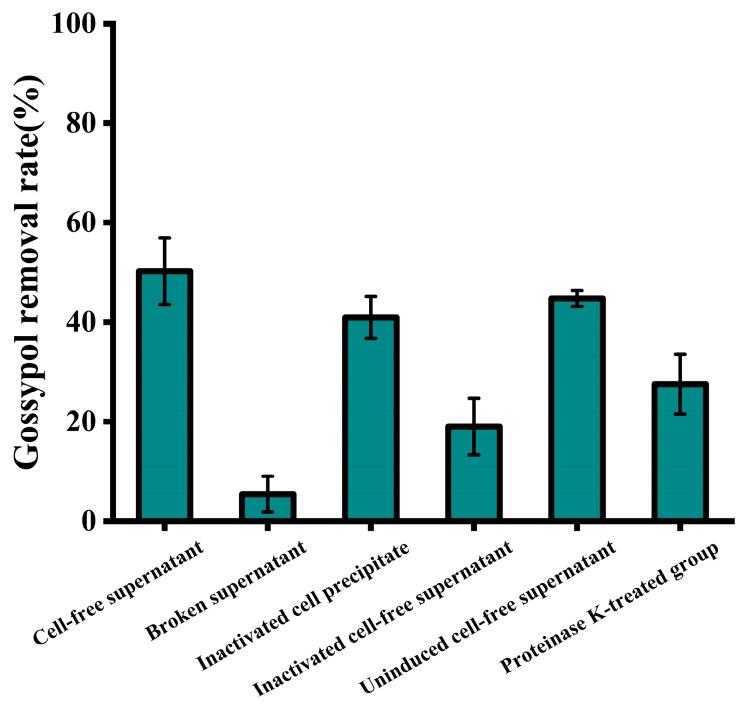
Intracellular and extracellular localization and identification of active components involved in gossypol removal by strain ZYS-3. Effect of different components and treated components of ZYS-3 on gossypol removal.

As shown in Figure 6, a notable difference in gossypol removal efficiency was observed between the intracellular and extracellular components of strain ZYS-3. Particularly, the gossypol removal efficiency of the cell-free supernatant reached 50.26%, surpassing the modest 5.4% removal observed with intracellular constituents, underscoring the prevalence of active agents for gossypol removal in the extracellular space. Additionally, the removal rates for gossypol by inactivated yeast cell precipitates and inactivated yeast cell supernatants were 40.92% and 18.99%, respectively. The physical adsorption of gossypol by inactivated yeast cell precipitates bears resemblance to the phenolic compound adsorption by Saccharomyces cerevisiae as elucidated by Razieh Sadat Mirmahdi et al. [20], where in the enhanced cell wall porosity post-heating facilitates robust adsorption of free phenolic compounds. The considerable reduction in gossypol removal rate in the supernatant of inactivated yeast cells substantiates the heat sensitivity of the extracellular active component, likely of a proteinaceous nature.

In the treatment group, a discernible reduction in the removal rate to 27.55% was noted in the supernatant upon the introduction of proteinase K. This observation indicates the presence of a gossypol-removing proteinase within the cell-free supernatant of strain ZYS-3, with the activity of this enzyme being compromised by proteinase K-induced structural alterations. Moreover, even in the absence of gossypol induction, the cell-free supernatant of strain ZYS-3 manifested a substantial 44.89% removal rate for gossypol. This finding underscores the strain’s capacity to spontaneously generate gossypol-removing (partial) enzymes without gossypol induction, demonstrating its inherent ability to naturally degrade gossypol.

### 3.7. Analysis of Intermediate Products of Gossypol Removal in the Supernatant of Strain ZYS-3

The Extended Ion Correlation (EIC) is a visual representation of ion intensities at specific mass-to-charge ratios over time. It serves as a pivotal tool in LC-MS analysis for determining characteristic ions, especially in the analysis of complex mixtures and trace analysis. In this study, the initial gossypol content introduced during fermentation was relatively low. Owing to the precarious nature of gossypol removal intermediates, their accumulation is challenging, resulting in notably diminished concentrations. Consequently, discernible peaks were absent in the total ion current (TIC) chromatogram of LC-MS. Therefore, in this experiment, leveraging molecular weight data acquired through comparative inquiries, the corresponding mass-to-charge ratios were employed to generate the mass chromatogram of the EIC extracted ion current chromatogram.

**Figure 7:**
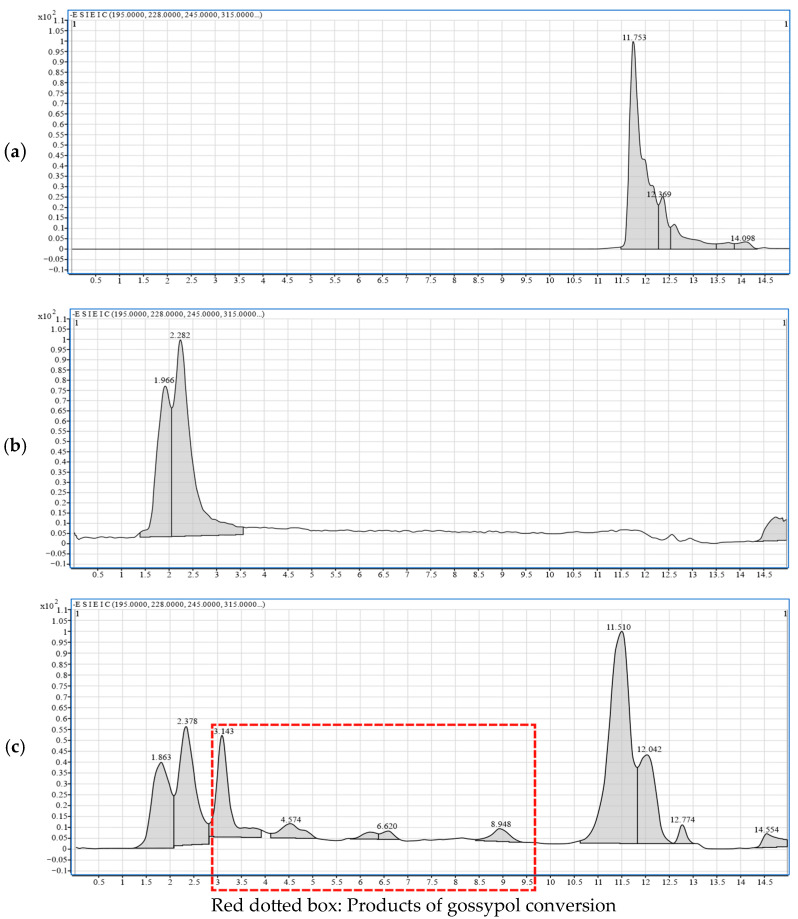
EIC extraction ion peak analysis of gossypol removal intermediates. (**a**) Gossypol standard sample. (**b**) YPD standard sample—supernatant of strain ZYS-3 without added gossypol. (**c**) Supernatant of strain ZYS-3 fermented with added gossypol for 6 h.

By comparing the chromatogram of the gossypol standard (Figure 7a) to that of the supernatant from cultures without gossypol (Figure 7b), it was determined that compounds eluting before 3 min likely originate from the culture medium. The peak observed at approximately 11.7 min, with an *m/z* of 517, was putatively identified as gossypol. Therefore, the new chromatographic peaks between 3 and 10 min in the experimental group (Figure 7c) are potential degradation intermediates of gossypol. Note that some products with structural similarities to gossypol may have similar retention times and could not be resolved by chromatographic behavior alone.

### 3.8. Analysis of Gossypol Removal Pathways

**Figure 8:**
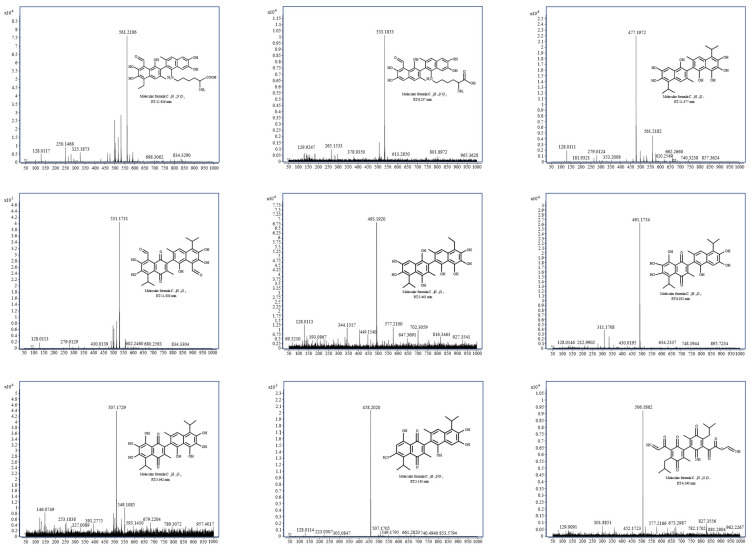
MS spectrum of intermediate products of gossypol removal.

Based on the identification results of the gossypol removal intermediate product in Figure 8, it is preliminarily speculated that the supernatant of strain ZYS-3 contains one or multiple enzymes proficient in gossypol removal. The specific removal mechanisms are divided into two pathways.

**Figure 9:**
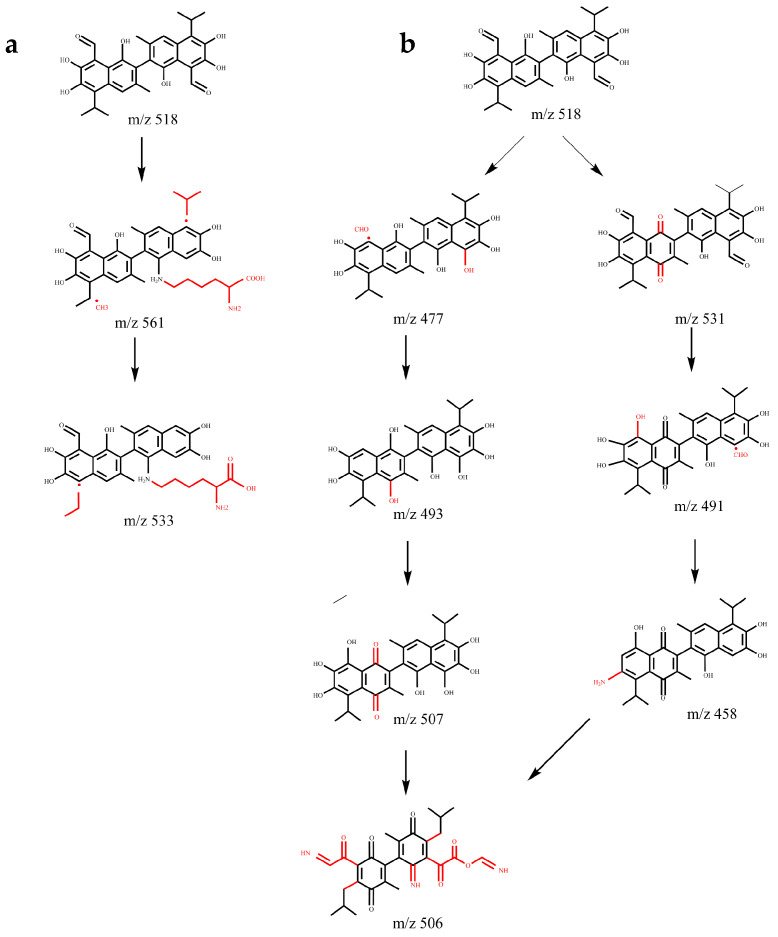
Gossypol removal pathways. (**a**) Lysine conjugation pathway. (**b**) Oxidation, elimination, and naphthalene ring opening pathways of active groups (phenolic hydroxyl group, aldehyde group).

Pathway 1: Gossypol-lysine conjugation pathway (Figure 9a). The ε-amino group of lysine readily engages in a Maillard reaction with the phenolic hydroxyl group of gossypol, leading to the formation of conjugated gossypol. The specific metabolic progression entails the initial loss of an aldehyde group by gossypol, followed by gradual hydrolysis of the tertiary group at the ortho position of the aldehyde group. Subsequently, one phenolic hydroxyl group on the benzene ring of gossypol binds with a lysine molecule, forming a compound with an *m*/*z* of 561. Finally, the alkyl side chain linked to the benzene ring undergoes continued hydrolysis, culminating in complete hydrolysis of the tertiary group at the ortho position of the aldehyde group, yielding a compound with an *m*/*z* of 533.

Pathway 2: Elimination, oxidation, and benzene ring opening of the active group of gossypol (Figure 9b). This pathway comprises two branches:

Branch 1: The initial step involves the elimination of the aldehyde group corresponding to the tertiary moiety of gossypol, leading to the formation of products with *m*/*z* 477 and *m*/*z* 493 upon hydroxyl addition. The introduction of the hydroxyl group increases the electron density of the naphthalene ring, facilitating the formation of quinone through oxidation of the phenolic hydroxyl group of gossypol, yielding the product with *m*/*z* 507. Subsequent removal processes entail the opening of the benzene ring of gossypol and its interaction with ammonium ions (NH_4_^+^) in the culture medium, ultimately forming the product *m*/*z* 506.

Branch 2: Through the elimination of the active phenolic hydroxyl group and the quinone formation reaction, the phenolic hydroxyl group is replaced by an amino group, yielding the product with *m*/*z* 458. Ultimately, the benzene ring undergoes ring opening, also producing the product with *m*/*z* 506.

## 4. Discussion

*M.guilliermondii*, an atypical yeast species, exhibits significant potential in enhancing the aroma of reconstituted tobacco leaves [21] and improving the aroma quality of dry red wine [22]. Studies conducted by the NAS Institute of Cell Biology in Ukraine indicate that, barring a few clinical isolates, the majority of *guilliermondii* strains are deemed safe for practical application [23]. In this research, an in vitro safety assessment was conducted in adherence to the Ministry of Agriculture and Rural Affairs’ Guidelines for the Identification and Safety Evaluation of Microbial Strains and Fermented Products Used in Direct Feeding. The results showed that strain ZYS-3 has no hemolytic activity, eliminating the risk of invasive pathogenicity. Moreover, its MIC values for nine commonly used antifungal drugs fell within the sensitive range, complying with resistance control criteria. Notably, safety assessments are rare in existing studies on gossypol-removing microorganisms, and the safety standard tests in this work provide a reference for the selection of feed strains.

Eliopoulos et al. conducted a fermentation study using a mixture of cottonseed meal and Lathyrus clymenum pericarp with *Pleurotus ostreatus*, resulting in an increase in protein content to 34.91%, a 5-fold enhancement in 6-β-glucans, a 26.71% reduction in lignin, and a 9-fold decrease in total gossypol content [24]. Similarly, Wang et al. fermented cottonseed meal with *Lactobacillus agilis* WWK129 for 5 days, achieving an 80% removal rate of gossypol. Additionally, neutral detergent fiber and acid detergent fiber contents decreased by 4% and 5%, respectively, while crude protein content increased by 4%, and the contents of most essential amino acids were significantly elevated [25]. Zhang Zhen Ting et al. [26] used *Bacillus coagulans* as the fermentation strain, conducting fermentation for 52 h at 40 °C with 15% inoculum, 2% corn flour, and 1% bran as additional carbon sources, resulting in an 81.33% gossypol removal rate. Liu Bifan et al. [27] utilized *Rhodotorula mucilaginosa* as the fermentation strain, fermented at 30 °C with 50% moisture content, and an 8% inoculum concentration for 11 days, achieving a gossypol removal rate of 73.29%. Chen Shengqin et al. [28] discovered that using acetic acid gossypol as the sole external carbon source, they isolated and screened a highly efficient gossypol-removing strain, *Rhodococcus erythropolis* RE-1. Under optimal fermentation conditions of 34 °C, 8 days, pH 5.0, a solid-to-liquid ratio of 1:0.5, and an inoculum concentration of 20%, the gossypol removal rate in cotton seed meal was determined to be 72.54%. To accurately assess the removal potential of strain ZYS-3, gossypol was intentionally supplemented to cotton seed meal to mimic actual detoxification scenarios. Under fermentation conditions of 30 °C, a fermentation time of 3 days, supplementation of 10% corn flour as an external carbon source, a cottonseed meal moisture content of 55%, and an inoculum level of 10%, ZYS-3 achieved a gossypol removal rate of 89.77%.

The gossypol removal activity test results indicated that gossypol removal is mediated by extracellular enzymes produced by strain ZYS-3. Previous studies have identified active enzymes involved in gossypol removal: Soares Neto et al. identified that the fungus *P. lecomtei* BRM044603 degrades gossypol in cottonseed meal by activating the secretion of oxidases and laccases [29]. Wang Weikang et al. elucidated, through transcriptomic analysis and gene knockout experiments, the pivotal regulatory roles of bifunctional acetaldehyde coenzyme A/alcohol dehydrogenase and catechol 2,3-dioxygenase in *Ruminococcus* during gossypol bioremoval [30]. Additionally, Chen Si et al. performed whole-genome sequencing of a gossypol-removing *Raoultella* sp. strain, annotating protein-coding genes such as aldehyde dehydrogenase, aldehyde oxidase, dioxygenase, and methyl hydroxylase that are potentially involved in gossypol removal [31]. Furthermore, Zhang Li et al. conducted transcriptomic analysis of tropical yeast ZD-3 under gossypol stress conditions, revealing a significant upregulation of the glutathione peroxidase-like peroxidase gene [32]. These enzymes primarily participate in redox and ring-opening reactions. In contrast, in our study, utilizing the extracellular enzymes secreted by strain ZYS-3 in the supernatant, we identified two primary metabolic pathways responsible for gossypol removal. One pathway involves binding with lysine, while the other involves the elimination, oxidation, and ring-opening of the active groups (phenolic hydroxyl and aldehyde groups) of gossypol [15,30]. Analysis of removal products showed a significant reduction in phenolic hydroxyl and aldehyde groups, coupled with naphthalene ring opening phenomena, indicating a potential reduction or elimination of toxicity in the removal products. However, the identification based on LC-MS remains putative. To definitively confirm the chemical structures of these intermediates, it is necessary to employ techniques such as nuclear magnetic resonance (NMR) spectroscopy alongside comparison with authentic standards. Furthermore, the practical toxicity of any residual compounds following gossypol removal must be evaluated by applying the ZYS-3-fermented cottonseed meal in livestock and poultry feeding trials. Additionally, future work will focus on utilizing proteomic and genomic approaches to identify the specific types of extracellular enzymes employed by strain ZYS-3 for gossypol degradation.

## 5. Conclusions

This study screened a highly efficient yeast strain, ZYS-3, identified as *Meyerozyma guilliermondii*, capable of removing gossypol from cotton field soil. Strain ZYS-3 exhibited non-hemolytic properties and susceptibility to nine commonly utilized antifungal drugs, indicating preliminary biosafety. In solid-state fermentation experiments using cotton ginning waste for gossypol removal, strain ZYS-3 achieved an 89.77% removal rate following optimization of the solid-state fermentation conditions. Strain ZYS-3 produces extracellular enzymes to degrade gossypol. LC-MS analysis conducted with these extracellular enzymes revealed two metabolic pathways for gossypol removal: one involving binding with lysine, and the other involving the elimination, oxidation, and naphthalene ring opening of active groups (phenolic hydroxyl groups and aldehyde groups).

## Figures and Tables

**Table 1:** Physiological and Biochemical Characteristics of Yeast Strain ZYS-3.

Indicator	Characteristics	Indicator	Characteristics
negative control	−	Sorbitol	+
D-Glucose	+	α-Methyl-D-glucose	+
Glycerol	+	N-Acetylglucosamine	+
Ketoglutarate	+	Fibro-disaccharide	+
L-Arabinose	+	D-Lactose	−
D-Xylose	+	D-Maltose	+
Calendula alcohol	+	D-Sucrose	+
Xylitol	+	Trehalose	+
Galactose	+	D-Pine trisaccharide	+
Inositol	−	D-Cotton seed sugar	+

+: Positive; −: Negative.

**Table 2:** Antimicrobial susceptibility testing results of strain ZYS-3.

Drug Name	Minimum Inhibitory Concentration (MIC)	Sensitivity Results
Amphotericin B	≤1	S
Flucytosine	Complete inhibition	S
Micafungin	≤0.125	S
Capreomycin	≤0.125	S
Fluconazole	≤4	S
Ethanol	≤0.25	S
Voriconazole	≤0.3125	S
Posaconazole	≤0.5	S
Itraconazole	≤0.5	S

S: Sensitive.

## Data Availability

The original contributions presented in this study are included in the article/Supplementary Materials. Further inquiries can be directed to the corresponding author.

## Supplementary Materials

The following supporting information can be downloaded at: https://www.mdpi.com/article/10.3390/microorganisms13102251/s1, Supplementary File: Sequence alignment results and the annotation of the 26S rDNA target region of *M guilliermondii* ZYS-3.
